# Piezoelectric Sensors as Energy Harvesters for Ultra Low-Power IoT Applications

**DOI:** 10.3390/s24082587

**Published:** 2024-04-18

**Authors:** Francesco Rigo, Marco Migliorini, Alessandro Pozzebon

**Affiliations:** Department of Information Engineering, University of Padova, 35131 Padova, Italy; francesco.rigo.3@studenti.unipd.it (F.R.); marco.migliorini.1@studenti.unipd.it (M.M.)

**Keywords:** energy harvesting, piezoelectric, vibration, Industrial Internet of Things, LoRaWAN

## Abstract

The aim of this paper is to discuss the usability of vibrations as energy sources, for the implementation of energy self-sufficient wireless sensing platforms within the Industrial Internet of Things (IIoT) framework. In this context, this paper proposes to equip vibrating assets like machinery with piezoelectric sensors, used to set up energy self-sufficient sensing platforms for hard-to-reach positions. Preliminary measurements as well as extended laboratory tests are proposed to understand the behavior of commercial piezoelectric sensors when employed as energy harvesters. First, a general architecture for a vibration-powered LoRaWAN-based sensor node is proposed. Final tests are then performed to identify an ideal trade-off between sensor sampling rates and energy availability. The target is to ensure continuous operation of the device while guaranteeing a charging trend of the storage component connected to the system. In this context, an Ultra-Low-Power Energy-Harvesting Integrated Circuit plays a crucial role by ensuring the correct regulation of the output with very high efficiency.

## 1. Introduction

The rapid growth of wireless sensing technologies in the Internet of Things (IoT) domain has made energy consumption a critical challenge. Many Wireless Sensor Nodes (WSNs) still rely on batteries, which require constant human intervention, increasing costs and limiting the scalability of many applications. To overcome this challenge, energy harvesting offers a promising solution by eliminating the need for frequent battery replacements, which reduces maintenance costs and improves the scalability of WSN deployments. This approach involves new technologies capable of scavenging small amounts of energy from various environmental phenomena, bringing the research closer to the world of renewable energies. One of the most significant advantages of this method is the ability to power electronic devices without relying on traditional sources like batteries or electrical outlets. This eliminates the need for constant human intervention, significantly expanding the potential application areas for these devices.

Due to the continuous growth of technologies in this field, new devices now offer the possibility to harvest energy from an increasingly wider range of sources, allowing for further scaling application scenarios of WSNs. This paper focuses on piezoelectric energy harvesting, which utilizes piezoelectric materials to convert mechanical energy into electrical energy. These robust and durable devices can generate electric charge from various sources, including vibrations in footsteps, buildings (like bridges), or industrial machinery, making them suitable for harsh environments. Overall, piezoelectric energy harvesting is a promising technology within the IoT domain and in industrial automation in particular: it can be employed for Industrial Internet of Things (IIoT) devices and may be used to power remote sensors by scavenging energy from industrial vibrations.

In this context, the aim of this study is to demonstrate the usability of low-cost off-the-shelf piezoelectric sensors as energy harvesters for ultra-low-power sensing applications. The industrial context proves to be ideal since in this scenario many vibration sources can be found, but the results presented in this paper are thought to be as general as possible, and thus also exploitable for applications to be deployed in other contexts any time there is the presence of vibrating items.

In the proposed experimental setup, piezoelectric transducers are combined with a dedicated power management integrated circuit (IC) to guarantee the energy independence of a generic low-power IoT application. In order to keep the outputs of the tests independent of the specific applications, only the core elements of a generic IoT node are integrated into the experimental setup, assuming that data transmission is in general the most power-hungry activity. Indeed, demonstrating the successful functionality of a generic low-power sensor node serves to illustrate the versatility of piezoelectric sensors across various application contexts.

Similar to numerous other distributed IoT systems, transmission is achieved by using the Long Range Wide Area Network (LoRaWAN) technology. The LoRaWAN protocol, which utilizes Long Range (LoRa) modulation, overcomes two critical requirements for IoT sensor nodes: minimal power usage and wide communication range. Consequently, it stands out as one of the most suitable telecommunication technologies among the Low-Power Wide-Area Networks (LPWANs).

In this context, this paper aims to investigate the possibility of using piezoelectric sensors for powering an ultra-low-power IoT node. The final goal is to define suitable conditions by establishing a proper balance between the data acquisition and transmission frequency and the charging capacity of the storage component integrated into the system. The rest of this paper is organized as follows: Section 2 explores related studies, whereas Section 3 elaborates on the system’s architecture. Section 4 examines the power consumption of the device. Laboratory tests are presented and discussed in Section 5 and Section 6, whereas Section 7 presents the conclusions.

## 2. Related Works

In recent years, energy harvesting has assumed a fundamental role in addressing the continuous growth of low-power applications [1]. A wide variety of alternative sources have been explored and adapted to supply many systems, enabling the deployment of new IoT nodes in increasingly remote scenarios [2].

Currently, harvesting energy from physical phenomena is a well-established technology, with many power sources such as heat, solar energy, or even wave motion extensively characterized. For example, authors in [3,4] provide detailed characterizations of thermoelectric generators (TEGs) and their utilization in highly energy-demanding IIoT applications. Their work demonstrates that, in the presence of a reliable heat source, these devices are capable of fully supporting a consuming application. Other authors, such as Olzhabay et al. [5], have described an indoor IoT sensor node based on perovskite solar cells, demonstrating their ability to power low-power applications even under conditions of very low irradiance. Bruzzi et al. [6] have investigated a typical LoRaWAN-based sensor node powered by dye-sensitized solar cells (DSSCs) under challenging conditions, showcasing the capacity of these devices to adequately supply power to the system even on cloudy days.

In addition to the advancements in IoT sensor nodes powered by well-known energy sources, there has been significant progress in the field of vibration energy harvesting. Vibrations are indeed common in many environments, such as in buildings, bridges, vehicles, and machines, and can be caused by various sources, such as traffic, wind, and the operation of machinery. Vibration energy-harvesting technology uses piezoelectric, electromagnetic, and electrostatic transducers or triboelectric nanogenerators to convert the mechanical energy of the vibrations into electrical energy. Electromagnetic vibration energy harvesters (EMEHs) [7] operate based on Lenz and Faraday’s laws, exploiting magnetic induction. By connecting a magnet to the vibrating source and allowing it to move within a coil, a current is generated proportionally to the variation in the concatenated flux of the magnetic field with the surface of the coil. Electrostatic vibration energy harvesters (ESEHs) [8,9] convert mechanical energy into electrical energy by exploiting the movement of electrons between the charged plates of a capacitor. One plate, connected to the vibrating source, induces relative motion with the other plate, causing a variation in total capacitance and leading to the generation of a static current. Triboelectric nanogenerators (TENGs) [10] rely on the triboelectric effect, which involves the generation of an electric charge when two dissimilar materials come into contact and then separate. TENGs exploit this effect by using materials with different triboelectric properties to generate electric charges through repeated contact and separation, typically induced by mechanical motion. TENGs can harvest energy from a variety of mechanical sources, including friction, vibration, and motion.

Piezoelectric harvesters (PEHs), as seen before, exploit the piezoelectric effect, which can be direct or inverse [11,12]. The direct one refers to the possibility to generate electric charges from mechanical stress or pressure, whereas the inverse one describes the ability of these materials to convert electrical energy into a mechanical one. The former is the one we are analyzing in this paper, whereas the latter is mostly used to generate sound waves (such as in buzzers and speakers). PEHs have been used in a variety of applications, like, for instance in [13], where the purpose was to power a wearable device exploiting upper limb movements. Furthermore, other parts of the body have been studied and proven efficient for these types of applications. As Shenck and Paradiso performed in [14], using specific piezoelectric materials to build shoes’ insoles, they were able to activate while walking with an RF tag, which transmitted a short-range wireless identification code. Indeed, as suggested in [15], a 68 kg man can indeed generate about 67 W by simply walking at a speed of two steps per second. Remaining in human body-related applications [16], medical devices like pacemakers have been integrated with flexible piezoelectric materials to extend their life.

A different application field is, for instance, the monitoring of buildings and infrastructures, like in [17], where the study was based on the generation of energy by applying piezoelectric material under a bridge, considering that when a car passes, a strain on the bridge is caused. Or again, Ref. [18] demonstrated the possibility of harvesting low-frequency vibrations obtained from the vehicles passing over speed bumps, and Ref. [19] analyzed a road energy harvester that could extract power from a roadway. A notable recent application is presented in the work by Dziadak et al. [20]. The authors developed a power supply system for a Wireless Sensor Network (WSN) node designed for monitoring the temperature of axle boxes and bearings in railroad wagons. To tackle the challenge of operating in harsh environments with limited power sources, they designed a piezoelectric energy harvester comprising three piezoelectric elements placed on a double-arm pendulum beam. Their research demonstrated the efficacy of a compact pendulum harvester, showcasing its ability to significantly extend the operational lifespan of the measurement node, even at low vibration frequencies. The literature, as referenced in [21], illustrates the different range of piezoelectric materials available and their mounting structures, highlighting the remarkable adaptability of these devices to various environments. Recent studies, such as [22], have explored the utilization of piezoelectric devices in wind energy harvesting, showcasing their versatility and potential applications in renewable energy generation. These research efforts evaluate the key factors influencing the performance of energy-harvesting systems and provide valuable recommendations for further optimization. The focus of the article is to highlight modeling techniques for Piezoelectric Wind Energy Harvesters (PWEHs), analyzing their structural functionality and assessing their efficacy in harnessing wind energy through phenomena such as vortex-induced vibration, flutter, and galloping. Additionally, this paper offers insights into future trends in PWEH development and discusses recent advancements in wind energy-harvesting strategies utilizing piezoelectric materials. It analyzes various piezoelectric materials addressing existing challenges in wind energy harvesting, outlines potential routes for future development, and provides recommendations for further research in this field.

The literature extensively covers various contributions in the field of piezoelectric energy harvesting. However, this paper introduces a more straightforward and potentially cost-effective method for energy harvesting compared to the systems typically discussed, such as the one outlined in [23]; whereas Ref. [23] utilizes a complex bimorph configuration with PZT material, this work focuses on readily available, single-layer PVDF sensors (LDT0-028K from TE connectivity), making the design more practical and potentially more affordable. The aim of this paper is to advance existing piezoelectric energy harvesters by showcasing the feasibility of utilizing sensor-purposed piezoelectric transducers, not traditionally considered energy generators, for energy-harvesting purposes. This novel approach enables the development of smaller energy harvesters compared to conventional ones, addressing the increasingly stringent limitations in size and cost for WSN applications. To further dive into the components employed in our design and the measuring setup, the subsequent section proposes a general overview of the system architecture.

## 3. System Architecture

**Figure 1:**
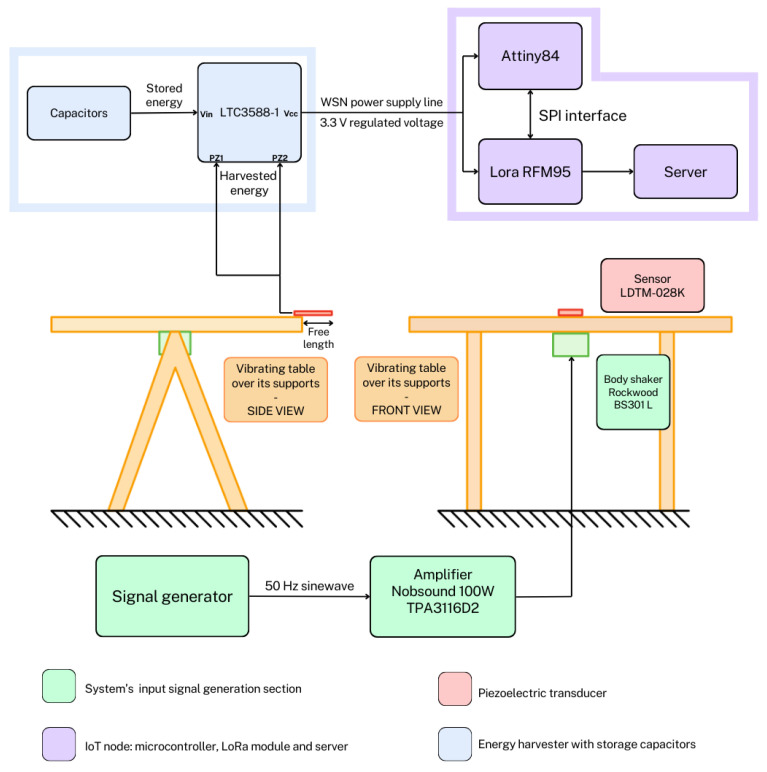
System architecture’s block scheme. The figure represents both the front and side views of the mounting setup. The signal generator outputs a 50 Hz, 1 V_*pp*_ sinusoidal waveform, which, after the amplification stage, reaches the body shaker. The latter generates a vibration that excites the piezoelectric sensor. Its harvested energy is processed and stored in capacitors. This allows to power the WSN.

This section provides a detailed analysis of the system architecture, outlining the functionalities of each component and their interconnections. The entire system architecture is outlined in the schematic diagram reported in Figure 1.

### 3.1. Vibrating Source and Harvesting-Related Architecture

**Figure 2:**
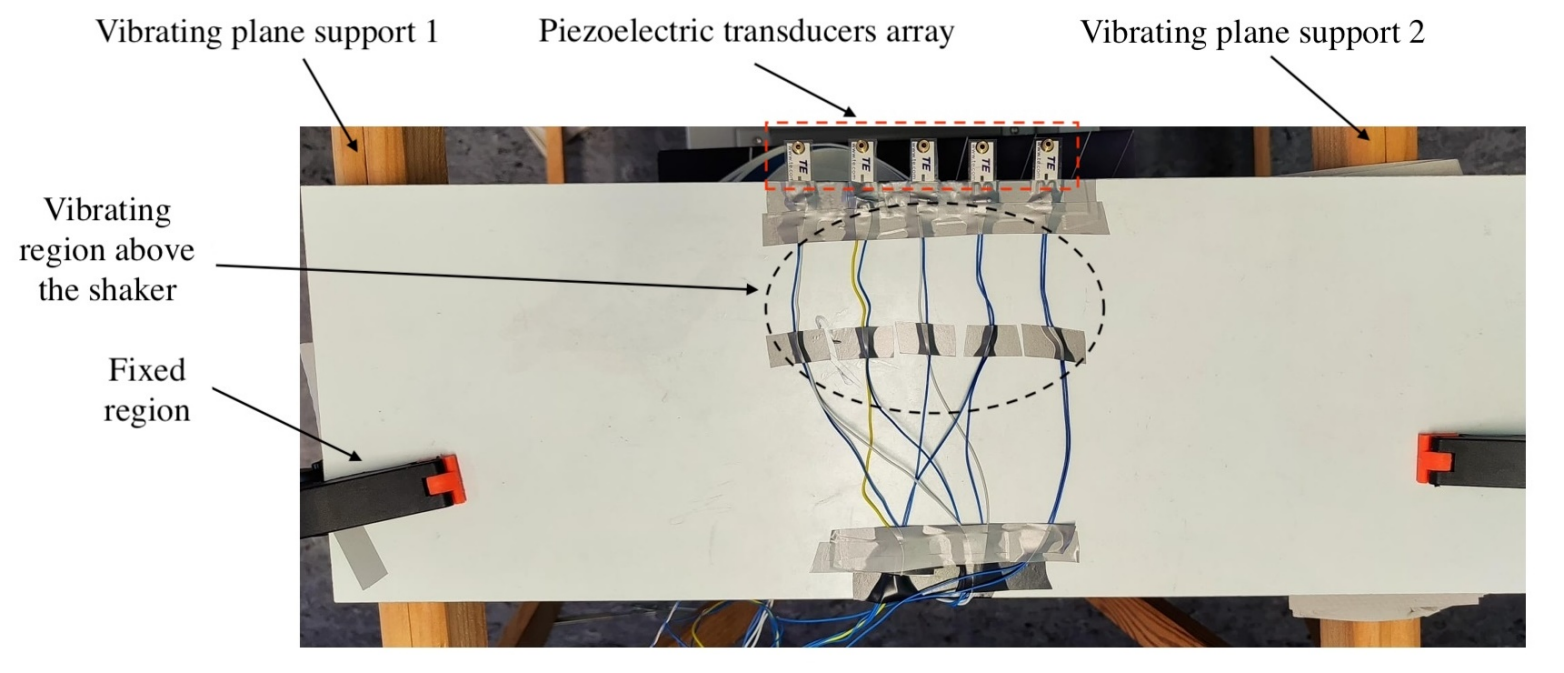
Top view of the vibrating plane with 5 piezoelectric transducers mounted. As better highlighted here, the vibrating region is the middle one, whereas the sides are fixed to the table supports.

**Figure 3:**
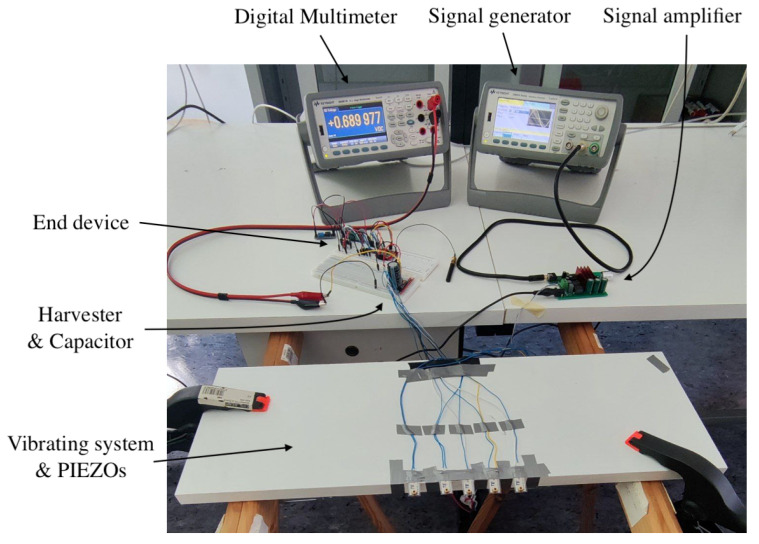
Complete setup: vibrating subsystem, signal generation and amplification subsystem, harvester and storage capacitor, end device with ultrasonic sensor embedded.

The system initiates with the generation of a controlled vibration using a signal generator, which produces a precisely defined 50Hz sine wave. This waveform is subsequently transmitted to a dedicated amplifier for signal amplification. The chosen amplifier model is the Nobsound TPA3116D2 based on the TPA31xxD2 series from Linear technology [24]. The amplified signal then drives the Rockwood BS301-L body shaker, which is mounted on the underside of a wooden table. Piezoelectric sensors, positioned on the top surface of the table in close proximity to the vibration source, are responsible for converting the physical vibrations into electrical energy. The chosen model for these sensors is the LDTM-028K [25]. This placement minimizes the potential for frequency alterations during vibration transmission through the wood. The central section of the table is designed to allow for free vibration, whereas the corners remain fixed to ensure stability and prevent unwanted resonances. Adhesive tape secures the piezoelectric sensors to the tabletop. Finally, the sensors are connected in parallel, and their combined electrical output, representing the harvested energy, is directed towards the energy harvester for further processing. Although a cantilever beam structure for energy harvesting is usually quite demanding in terms of needed space, the used transducers have very small dimensions, being 13 mm × 25 mm. In the system proposed in this paper, as a result of a trade-off between the generated power and the occupied volume (which could be important in a remote sensing node), five sensors have been mounted over the wooden table as better highlighted in Figure 2. The complete subsystem is shown in Figure 3, highlighting the shaker and piezoelectric sensors.

The outputs of the piezoelectric sensors, which generate sinusoidal signals at the same frequency as the vibration signal, are connected to the PZ1 and PZ2 inputs of the energy harvester. The energy harvester is a pre-built board that incorporates the Analog Devices chip LTC 3588-1 [26]. This component proves ideal for extracting energy from sources such as vibrations (piezoelectric) or AC power. It transforms incoming AC signals into DC for storage, regulates the output voltage to a selectable level, and employs a nanopower, highly efficient synchronous buck regulator. The harvester’s input stage incorporates an internal full bridge rectifier, generating a DC voltage that charges the storage capacitor connected to the $V_{in}$ pin. This DC voltage is internally clamped at 20 V and controls the UVLO (Under Voltage Lock Out) section, which determines when the buck should be enabled or disabled.

**Figure 4:**
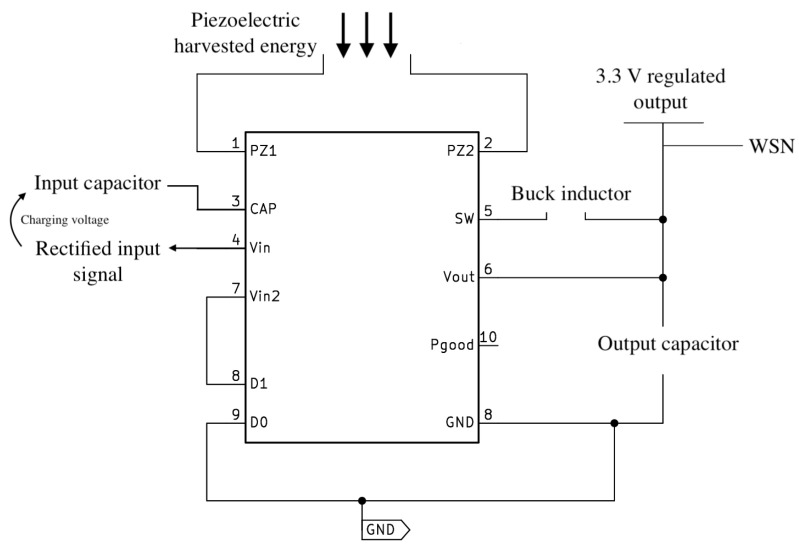
Internal schematic of the proposed harvester [26]. Numbers correspond to the ports of the components according to its physical layout.

A noteworthy feature of the harvester is the use of a hysteretic voltage algorithm to regulate the output, utilizing internal feedback from the $V_{out}$ sense pin. In this process, the output capacitor is charged beyond the desired regulated voltage through the inductor connected to pin SW, as depicted in Figure 4. The PMOS switch is turned on during this charging phase, enabling a gradual current increase up to 260 mA (dependent on input voltage, output voltage, and inductor value). Once the voltage slightly exceeds the regulation point, the PMOS switch is turned off. Under load current, the output capacitor discharges until the voltage falls below the regulation point. At this point, the regulator activates, and the NMOS switch is turned on, facilitating energy flow from the inductor to the output capacitor to maintain the desired output voltage. This cycle efficiently repeats during light loads due to minimal losses associated with hysteretic control.

During periods of inactivity in regulation (i.e., when the output capacitor supplies the load current), the buck converter enters a sleep state tracked by a sleep comparator. In sleep mode, the NMOS switch remains on even when the inductor current reaches zero, preventing unnecessary losses that would occur if the current freewheeled through the body diode of the NMOS. One of the most important components is the storage element to be used in conjunction with the harvester. On the input side of the IC, we have connected a large capacitor between VIN and GND to store excess energy generated by the piezoelectric sensors. Due to the specific characteristics of this harvester, it is not feasible to use a standard rechargeable battery. The circuit has a hysteresis threshold that requires the input voltage to exceed 5V before regulating the connected load. This voltage threshold is not compatible with a standard 3.7 V Li-Po battery and prevents the rapid discharge of the storage component by delaying the regulation of the output until sufficient energy has accumulated on the input side.

Considering the overall system efficiency, although the buck converter consumes more power during active switching, this duration is very short compared to the sleep time when harvesting energy. This design proves ideal for applications like the one in this paper, where the energy source provides small amounts of power.

### 3.2. Wireless Sensor Node’s Architecture

The harvester’s generated power serves as the energy source for the IoT node. The sensor node comprises two primary components: a Microcontroller Unit (MCU) and a LoRaWAN module. A basic LoRaWAN network is established between the end node and a private gateway positioned a short distance from the measurement location. The design incorporates an RFM95 LoRaWAN transceiver from HopeRF [27], facilitating transmissions at very low power levels. To manage the LoRaWAN module, an ATTiny84 MCU was selected due to its advantageous balance between simplicity, power consumption, and performance. In this application, no specific sensors were integrated into the system, assuming that the periodic transmission bursts dominate the overall power consumption. However, this concept will be further analyzed in subsequent sections, where an HC-SR04 ultrasonic sensor will be integrated into the system to demonstrate the versatility of the application when utilizing standard, low-energy-demanding sensors.

As mentioned earlier, the output voltage of the harvester can be adjusted to meet specific requirements. Despite the fact that both the MCU and the LoRaWAN transceiver can operate with a supply voltage of 1.8 V, we have set the output voltage to 3.3 V in our setup. This ensures compatibility with the MCU, LoRaWAN module, and a wide range of sensors available in the market. This voltage level is regulated by the buck converter inside the harvester, which maintains the output voltage at 3.3 V once sufficient energy is stored in the input capacitor. It is important to note that this regulation is independent of the voltage across the piezoelectric transducers’ electrodes. The voltage across the electrodes affects only the amount of energy harvested from the environment and, consequently, the charging speed of the input capacitor, but not the output regulation level. Theoretical analyses subsequently assume a standard voltage of 3.3 V as a result. The complete setup, including the measuring subsystem, is reported in Figure 3. In the next section, these theoretical aspects will be used to further analyze the power consumption of the components present in the design.

## 4. Power Consumption Analysis

Similar to many other low-power applications, the operation of the device has been divided into two main phases: an active phase where the system queries the sensors to collect new data and transmit them, and a power-down phase during which harvested energy is stored in the input capacitor to support subsequent transmission bursts. Ensuring the proper functioning of the device requires a careful analysis of both phases, as this enables optimization of both firmware and hardware, resulting in optimal performance in terms of maximum transmission rate. To do so, by using a 6.5-digit Keysight Technologies (Santa Rosa, CA, USA) 34461a Digital Multimeter and a Keysight Technologies DSOX3024T oscilloscope, we tested the current drawn by all the components during both the operating modes of the device. The results are summarized in Table 1, which presents the currents drawn during both the operating modes and the active time of each component.

The results clearly highlight that the transmission burst represents the main contribution to the power consumption during the device’s active phase. This paper’s objective is indeed to demonstrate the operational capability of a versatile IoT application integrating standard sensors. In this setup, the power consumption of these sensors remains relatively low compared to the peak energy demand observed during a transmission burst. It is important to notice that whereas our focus is on scenarios with low-power sensors, similar behavior can be expected in applications requiring high-energy-demanding sensors. However, analyzing power consumption in such instances necessitates accounting for these distinct factors.

Laboratory tests were carried out with a spreading factor (SF) of 7 and a bandwidth (BW) of 125 kHz. These settings enabled transmissions with data lengths ranging from 1 to 6 bytes to be completed in approximately 50 ms. Additionally, to effectively manage power consumption, the transmission power of the LoRaWAN transceiver was set to 2 dB. This level is typically adequate to cover distances of a few hundred meters that are well aligned with many industrial environments. By using these configurations, significant reductions in device power consumption were achieved, thereby facilitating the charging of the capacitor integrated into the system.

**Figure 5:**
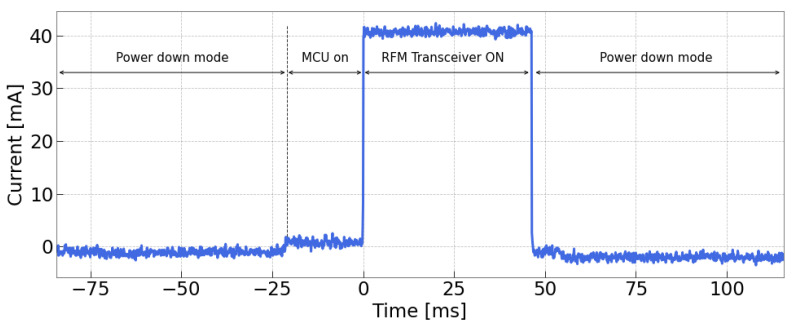
System power consumption during the active phase when no sensors are employed.

Figure 5 illustrates the operational cycle of the device in a scenario where no sensors were integrated. As depicted, the operation alternates between two distinct modes: a power-down mode, where energy consumption is minimized to facilitate the charging process of the storage capacitor, and an active phase where both the MCU and LoRaWAN module turn on to execute a transmission. During the active phase, the device undergoes a sequence of events. Initially, there is a phase lasting approximately 20 ms where only the MCU operates at very low power consumption to initialize the transceiver. Subsequently, a peak current of 40 mA is observed, lasting almost 50 ms, signaling the completion of the transmission burst. Conversely, in the sleep mode, both the MCU and LoRaWAN module transition into a power-down routine, significantly reducing consumption to just a few hundred nanoamperes.

Although the nanopower buck converter’s datasheet indicates minimal parasitic terms, it is worth briefly discussing these factors more in detail. The results from testing the nanopower buck converter are presented in the second row of Table 1. Here, the measured current reflects residual parasitic terms within the converter, requiring, in any case, careful examination as even minor currents could significantly impact the available energy. Indeed, during the piezoelectric vibration, we observed a current flow ranging from 10 μA to 14 μA into the input capacitor. According to the datasheet, the ultra-low quiescent current undervoltage lockout (UVLO) feature sets the harvester in two operating modes. When low energy levels are detected at the input, the buck converter shuts off, resulting in a loss of load regulation and 0.2μA draw. Conversely, if the input voltage exceeds the 5V threshold, the buck converter regulates the output voltage to the programmed level, activating the load. In this scenario, under open load conditions, a parasitic term of approximately 1μA is drawn from the output capacitor. During the active phase, the converter’s efficiency also plays a role, although this aspect will be further explored in the subsequent section. For what concerns the parasitics of the harvester, we considered the current draw to be 1μA given that in an ideal scenario the output never loses the regulation state during both the active and sleep mode of the device.

**Figure 6:**
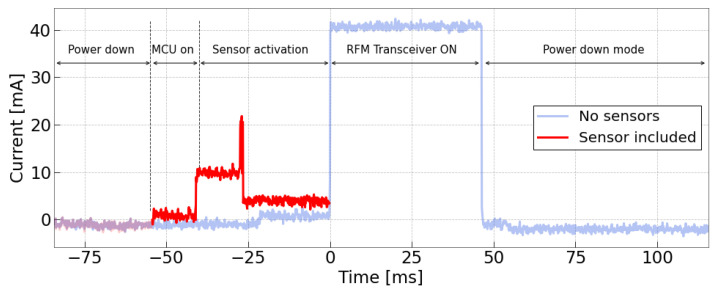
System power consumption during the active phase when an ultrasonic sensor is included in the system.

The second test campaign was conducted by integrating an HC-SR04 ultrasonic sensor into the system to assess the impact of a relatively power-hungry sensor on device power consumption. The last row of Table 1 reports the corresponding values tested for this sensor. To meet the sensor’s voltage requirement of 4 V to properly operate, a 5 V DC-DC step-up converter was added to the system. However, this setup served as an additional way to demonstrate the device’s adaptability under more demanding conditions induced by the increased energy consumption of the step-up converter. During the active phase, the sensor exhibited an average current of 9mA, with a peak reaching 23mA during the measuring phase. An additional BJT transistor was used to manage the sensor’s activation and deactivation, effectively minimizing its power consumption to negligible levels during sleep mode. A sample of the activation of the device is depicted in Figure 6. Here, only the sensor’s activation is superimposed on the previous trace, as the transmission burst remained almost identical regardless of whether the sensor was present. The sample shows that even with the integration of a standard sensor into the system, the transmission burst continues to dominate the energy consumption of the device, as evidenced by the integral of the previous plot, and whereas the inclusion of a sensor may marginally reduce the capacitor’s charging speed, the overall performance remains consistent in a general context. Consequently, subsequent tests are conducted with the sensor excluded from the system, thereby maintaining a more comprehensive overview of the application. This approach ensures adaptability to various environments and working conditions. Thanks to these achievements, further testing was possible. In particular, the following paragraphs report few considerations about the piezoelectric devices used here, a set of sizing equations and laboratory results performed to test the operation of the system.

## 5. System Tests

### 5.1. Piezo Mounting Considerations

This paper investigates the potential of the LDTM-028K piezoelectric transducer (TE Connectivity) for energy-harvesting applications. It is typically used as a sensor for triggering circuits or for acceleration measurement. The datasheet suggests the transducer may be better suited for detecting impacts, static pressure changes, or vibrations rather than energy conversion. However, this research explores its potential in the latter application. For optimal performance, mounting considerations need to be conducted. The resonant frequency of the transducer is indeed highly dependent on the applied tip mass and on the “free” length after the clamped section. The chosen model incorporates a 0.72 g tip mass to adapt the sensor to lower frequencies.

**Figure 7:**
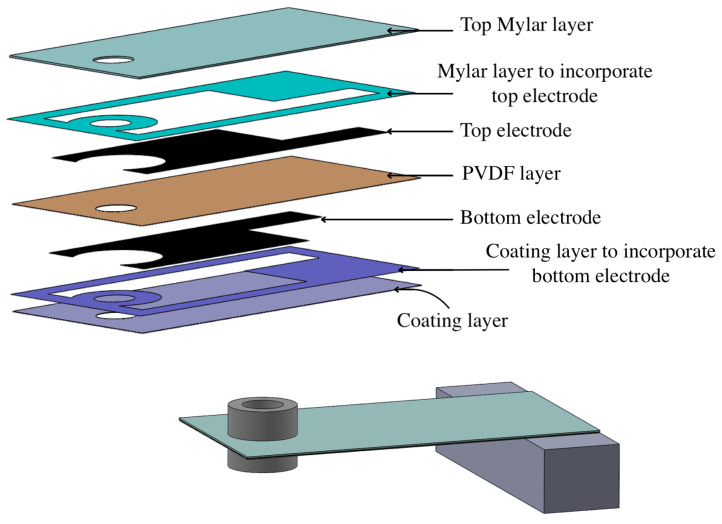
Piezoelectric transducer (LDTM-028K) layers stack.

The datasheet lacks information on the relationship between the “free” length and the resonant frequency of this transducer, which is therefore derived in here through the FEA (Finite Element Analysis) software ANSYS Student 2024 R1. In this way, it is shown how, through simple mounting considerations, these sensors can adapt to broad frequency ranges and how, in every condition, they are able to generate the necessary energy to power the considered IoT node. A 3D model of the piezoelectric transducer was first created in Solidworks using information from the datasheet. Unluckily, the manufacturer has chosen not to disclose certain specifications, such as thicknesses and material properties, in the datasheet. Consequently, the model was constructed using the given knowledge and has undergone multiple revisions to achieve the closest possible resemblance to the actual sensor. The final structure is reported in Figure 7 along with Table 2, which describes the mechanical parameters of each one of the component’s materials.

Since the 3D model, as previously mentioned, does not fully correspond to the real structure because of the missing details, there was the need to validate it through a simulation. This simulation compared the resonant frequency of the first mode with the commercially available version of the transducer from TE Connectivity LDT0-028K (without tip mass) for which the resonant frequency dependence on “free” length is provided.

**Figure 8:**
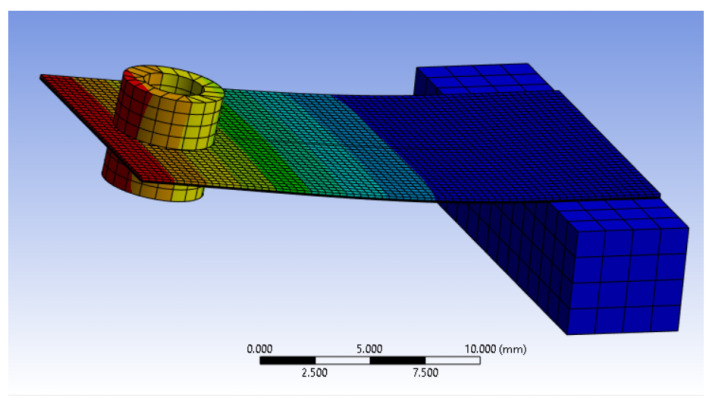
First mode shape of the LDTM-028K piezoelectric transducer (maximum and minimum displacements are shown respectively in red and blue).

The ANSYS model comprises a stack of various solid elements. Starting from the base, there is a coating material that extends onto the sides of the bottom electrode, effectively incorporating the electrode within the external coating. Next is the PVDF material layer, upon which the top electrode is printed. Surrounding all the sides and the top of the top electrode layer is a 5 mil Mylar layer. The exploded view of this structure (with tip masses omitted for image clarity) is depicted in Figure 7. Each layer is connected to its nearest layers through a bounded connection type. To mesh the structure, a body sizing of 0.35 mm has been selected specifically for this material’s stack. The mesh density can be seen in Figure 8.

**Figure 9:**
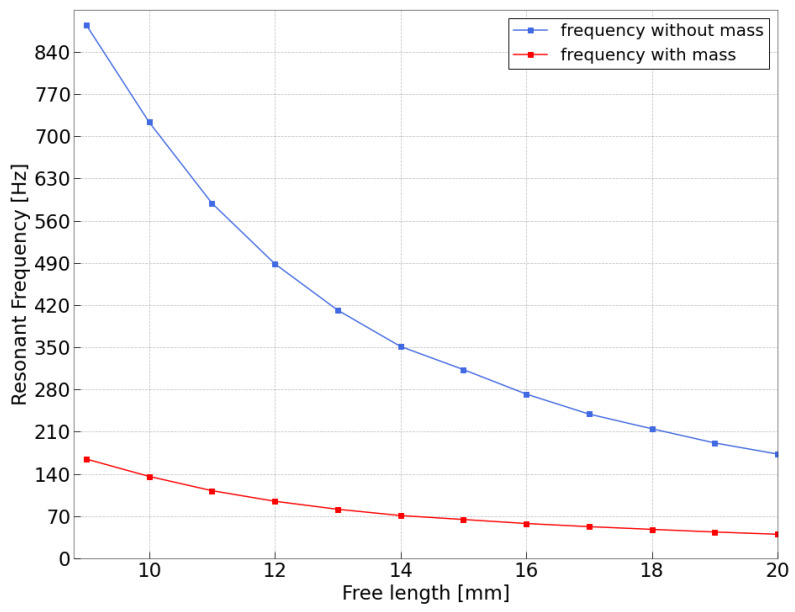
Simulated relation between resonant frequency and “free” length for the Piezoelectric transducers. The blue trace reports results when no tip mass is used, whereas the red trace represents the case in which 0.72 g mass is applied.

The results showed good agreement, with the resonant frequency starting at f = 180 Hz for the maximum “free” length (20 mm) and increasing exponentially towards higher frequencies. There was a slight discrepancy at high frequencies, where even minor model variations could lead to significant errors. This is likely due to the lack of detailed information about material properties, thicknesses, and their specific characteristics in the datasheet. However, the validation process proved beneficial. It allowed for the subsequent simulation of the device with the added tip mass. Both the simulation results are shown in Figure 9.

As expected from the datasheet, the resonant frequency for around 0.7 g tip masses is about 40 Hz. Reducing the “free” length of the cantilever beam structure, the resonant frequency increases. For the application of this paper (i.e., sinewave at 50 Hz), the best mounting configuration can be expected to have a “free” length of around l=17 mm. Figure 8 shows an ANSYS environment image, detailing how the first mode shape appears for the chosen cantilever beam structure.

From a mathematical perspective, the same results can be derived according to the Euler–Bernoulli beam theory. Indeed, referring to the equations that rule free vibrations, it is possible to derive analytically the modal frequencies of a cantilever beam. Of course, it is necessary to impose the boundary conditions (i.e., where it is fixed and where it is free) and then solve the resulting equation. It is important to derive the second moment of inertia (or area moment of inertia) of the cantilever with respect to its neutral axis and to know its elastic modulus. Since in this paper there is a stack of materials with different moments of inertia and different elastic moduli, the structure has been approximated as a rectangular parallelepiped with a thickness equalling the sum of all the thicknesses of the layers, which is equal to t=205μm. Moreover, the elastic modulus was considered equal to E=9 GPa, which is slightly lower than that of the highest stack to also consider the less rigid layers.The equation that determines the displacement for each mode for a beam without tip mass is as follows:

(1) $$w_{n}=A\cdot cosh\left(\beta_{n}x\right)+B\cdot sinh\left(\beta_{n}x\right)+C\cdot cos\left(\beta_{n}x\right)+D\cdot sin\left(\beta_{n}x\right)$$

The value of $\beta_{n}$, where *n* refers to the *n*th mode, is given as follows:

(2) $$\beta_{n}={\left(\frac{EI}{\mu \omega_{n}^{2}}\right)}^{4}$$

Therefore, solving Equation (1) after imposing the boundary conditions, it is possible to derive the values of $\beta_{n}$ and to finally extract $\omega_{n}$, given that *E* is the cantilever elastic modulus, *I* is its second moment of inertia, and μ is the beam’s mass per unit length.

The second moment of inertia of the beam was calculated as the following:

(3) $$I=\frac{1}{12}bt^{3}$$

where *b* is the beam width and *t* its thickness.

Imposing the boundary conditions and solving the above written equations, the first resonant frequency is found to be equal to the following:

(4) $$f_{1}=\frac{3.516}{2\pi L_{free}^{2}}\cdot \sqrt{\frac{EIL_{tot}}{m_{tot}}}$$

where $L_{free}$ is the free length, $L_{tot}$ is the total cantilever length (equal to $25.4\cdot 10^{-3}$ m), $m_{tot}$ is the total mass (equal to $1.297\cdot 10^{-4}$ kg), *E* is equal to 9 GPa, and *I* results equal to $9.33\cdot 10^{-15}$ m^4^.

The results obtained through this final equation are almost identical to the ones obtained through simulation for the LDT0-028K sensor, and allow a better understanding of how ANSYS solves the modal analysis.

### 5.2. Preliminary Tests

To initiate preliminary tests, the sizing of the main components was determined based on the evaluation of power requirements and energy availability. First of all, the input capacitor was sized, exploiting the loading currents reported in Table 1. From the power consumption analysis, the total energy required by the load during a transmission burst can be calculated following Equation (5), where $V_{out}$ represents the buck converter output voltage, $I_{load}$ is the average current draw during a burst and Δt is the total active time required to complete a transmission.

(5) $$W_{n}=V_{out}\cdot I_{load}\cdot \Delta t$$

In a similar way, we can express the energy variation on the input side as described in Equation (6), where $\Delta V_{in}=V_{in2}-V_{in1}$ is the voltage drop observed across the capacitor $C_{in}$ when subjected to an energy variation $\Delta W_{in}$

(6) $$\Delta W_{in}=\frac{1}{2}\cdot C_{in}\cdot \left(V_{in2}^{2}-V_{in1}^{2}\right).$$

Considering that $V_{out}$ was configured to 3.3V, looking at the harvester’s datasheet reveals the hysteresis cycle of the UVLO, with $V_{UVLO,1}=4\;V$ and $V_{UVLO,2}=5\;V$. To prevent the load from shutting off, the maximum voltage drop across the input capacitor should be less than the hysteresis amplitude (1V). This implies that the energy variation resulting from load activation, factoring in the buck converter’s efficiency (approximately η≈75%), must be within the allowed energy variation at the input side. Furthermore, it is important to notice that the sizing equations are derived by using the worst-case condition. Indeed, from the harvester’s datasheet, the efficiency is guaranteed to be at least greater than η=75%, possibly up to 90%. Given that these results are directly provided by the National Instruments harvester, additional tests into this aspect were considered redundant. The following equation can therefore be used to properly size the capacitor.

(7) $$W_{n}\leq \eta \cdot W_{in,max}=\eta \cdot \left[\frac{1}{2}\cdot C_{in}\cdot \left(5^{2}-4^{2}\right)\right]$$

From which the final capacitance value was estimated to be as follows:

(8) $$C_{in}\geq 2\cdot \frac{V_{out}\cdot I_{load}\cdot \Delta t}{(5^{2}-4^{2})\cdot \eta }\Rightarrow C_{in}\geq 2.74\;\left[\mathrm{mF}\right]$$

The values of $I_{load}$ and Δt were determined based on the characteristics observed in the transmission burst reported in Figure 5. To provide some margin of safety, we calculated Δt, assuming a value of 70 ms, under the assumption that the entire load activation occurs at the maximum current draw. To be safe, $C_{in}=4.7\;\mathrm{mF}$ was used, resulting in a slightly oversized capacitor.

Having determined a suitable capacitor size, we then needed to consider the overall power consumption of the system to establish some preliminary considerations about transmission timings. This helped in determining the minimum transmission rate which could guarantee a correct system’s functioning. Indeed, it is possible to express the voltage variation across the input capacitor as follows:

(9) $$\Delta V_{in}=\frac{1}{C_{in}}\cdot \int_{t_{0}}^{t_{0}+T_{x}}i_{in}\left(\tau \right)\;d\tau$$

By recalling that at the input a constant current equal to 14–15 μA was tested and that when the load is sleeping 5μA are absorbed at the output, the total net current flowing into the input capacitor is given by the following:

(10) $$I_{in}=14\;\mu A-\frac{5}{\eta }\;\mu A=7.33\;\mu A$$

From which, by recalling that in a worst-case condition the activation of the load causes a voltage drop equals to the amplitude of the hysteresis cycle, it follows that:

(11) $$T_{x}=\frac{C_{in}\cdot \Delta V_{in}}{I_{in}}\Rightarrow T_{x}\approx 641\;s$$

Therefore, in a worst-case condition, where the load causes a voltage drop of 1 V, a transmission period of 10 min should be large enough to charge the input capacitor to a level sufficient to sustain a new transmission burst. However, laboratory tests showed that the activation of the load never causes a voltage drop larger than 0.4–0.5 V; therefore, we were also able to test higher transmission rates.

**Figure 10:**
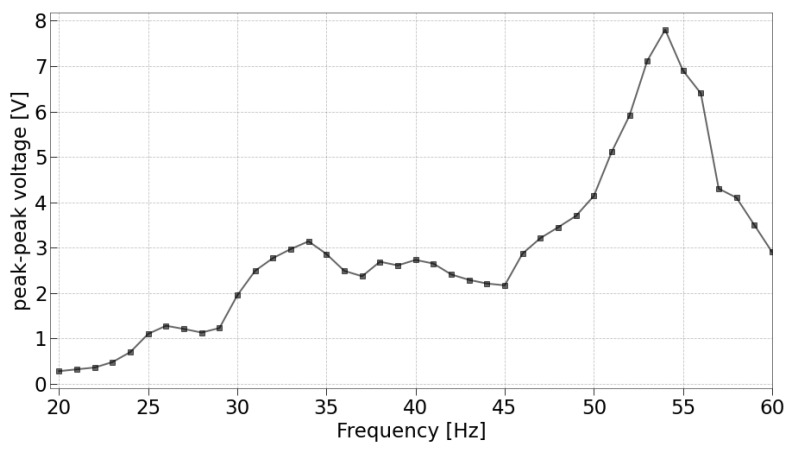
Tested peak-to-peak voltage for the piezoelectric device clamped at 17 mm.

As a final preliminary test, a characterization was conducted to examine the peak-to-peak voltage versus frequency, given a fixed free length of 17 mm, aiming to validate its proximity to the simulation expectations. The resulting plot is illustrated in Figure 10.

Several noteworthy observations can be made regarding this plot. First, it is evident that the curve does not resemble a ‘Gaussian’ shape, but rather exhibits various small peaks for lower frequencies than the resonant one. This deviation from the expected shape may be attributed to the experimental setup utilized in this study, which involves a wooden table as the medium for vibration propagation. The table likely possesses internal resonances and may respond differently to various stimuli. Consequently, it is possible that the vibration reaching the base of the piezoelectric transducer undergoes slight frequency alterations under different excitations. Such characteristics are reflective of more realistic environments, where perfectly sinusoidal vibrations generated by a shaker are unattainable.

Although the structure may indeed introduce some alterations to the characteristic, the primary peak remains distinctly visible and occurs around 54 Hz. This finding deviates slightly from the simulation outcome, which yielded a resonant frequency of $f_{res}=52.629$ Hz for a 17 mm free length. The minor variance can once more be attributed to the approximations inherent in the 3D model.

However, rather than focusing solely on the best-case scenario, which is often challenging to replicate in real-world environments, the subsequent section deliberately analyzes the system’s power consumption and its charging trends under a 50 Hz vibration. Given that the peak-to-peak voltage at 50 Hz is significantly smaller compared to the resonant frequency one, assessing the system’s ability to harvest sufficient energy at this frequency is crucial. If the system can indeed achieve satisfactory energy harvesting at 50 Hz, it suggests that piezoelectric sensors such as the LDTM-028K can be effectively utilized even without perfect tuning to the resonant frequency. These tests demonstrate the sensors’ capability to provide adequate energy in different environments, highlighting their practical value in real-world applications.

### 5.3. Power Consumption Tests

One last series of tests was conducted to analyze the overall behavior of the system, including both the transmission subsystem and the harvesting component. The final goal was to observe the device’s operation over extended working periods to establish whether the piezoelectric transducers could ensure energy self-sufficiency for a given transmission rate. To evaluate the device’s behavior, a Keysight Technologies 34461A Digital Multimeter was configured to sample the voltage across the input capacitor. This specific multimeter exploits an internal memory capable of storing samples for observation periods lasting up to two hours. Hence, this feature was used to test the system over longer observation intervals. The tests were carried out after allowing sufficient time for the vibrations of the vibrating plane to stabilize. However, as observed in the subsequent voltage trends, minor fluctuations persist over extended periods, resulting in variable charging rates. Nonetheless, the objective of this study is to demonstrate that properly excited piezoelectric transducers can support a general-purpose IoT application. Thus, as long as a positive trend is evident across the storage capacitor, the design specifications are met, despite the lack of constancy over time. As previously mentioned, to face the low currents flowing into the input capacitor, five piezoelectric sensors were connected in parallel. Their compact size enables the placement of multiple piezos on the same vibrating surface without significant space wastage, thereby increasing the energy harvested and allowing for heavier load conditions.

**Figure 11:**
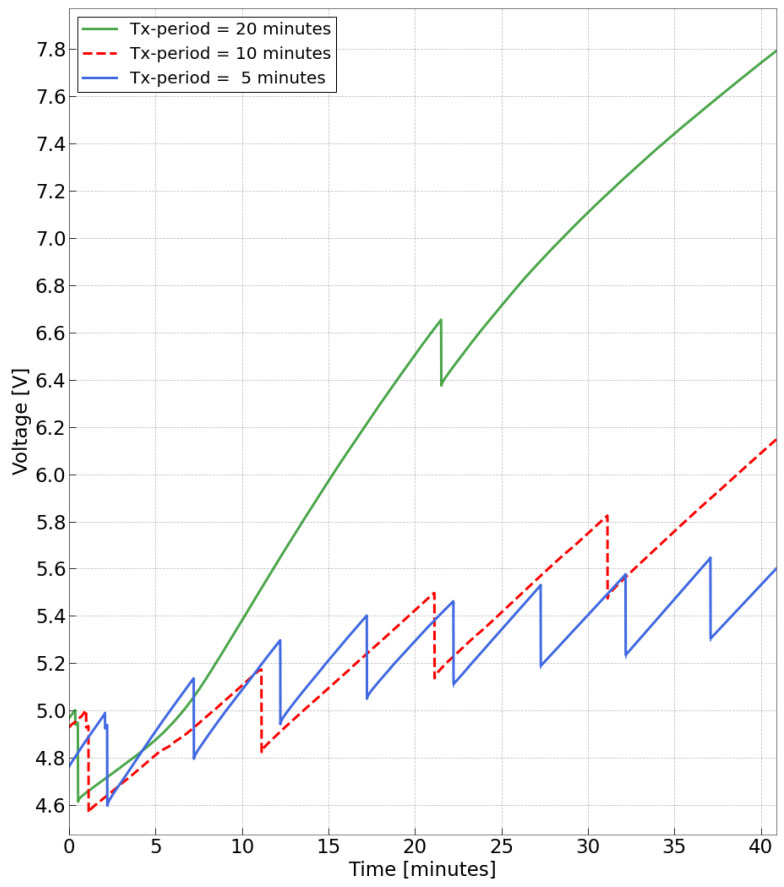
Capacitor voltage trend for different transmission rates over 40-min observation interval.

An initial transmission period of 20 min was employed, and from this starting point, the system was pushed into progressively more demanding conditions by increasing the transmission rate. The MCU unit and the LoRaWAN module were programmed to wake up only for the time necessary to complete the packet transmission. All trends are depicted in Figure 11, where all traces are overlapped to facilitate comparison.

Notably, focusing on the green trace, which represents a transmission every 20 min, the downward peaks align with RFM95 transceiver activation. Although these peaks decelerate the charging process, they do not significantly impact the overall trend. At the end of the 40-min observation interval, there remains a margin of almost 3V. As previously noted, the charging speed gradually slows towards the end of the observation period, attributed to variations in the vibrating plane and the decreased performance of the power amplifier due to heating. Although the introduction of a few cooling fans mitigated the latter effect, since the overall trend remains positive, further investigation into this aspect was not necessary.

Following the observed margin in the initial test, the transmission rate was incremented to one transmission every 10 min, and subsequently, to a radio transmission every 5 min. The voltage trends obtained are depicted in Figure 11 in red and blue. Once more, a positive trend was noted, with an overall voltage gain inversely correlated with the transmission rate. Focusing on the first transmission observed in all the traces depicted in Figure 11, an important observation arises. It is observable that the charging slope before the first transmission is slightly higher than the slope immediately following the initial downward peak. This observation aligns with the theoretical analysis, as before the first peak, the buck converter has not yet reached regulation, and thus, no load is connected to the system. Conversely, when the input voltage crosses the input hysteresis threshold, the load reaches regulation status, initiating the first transmission and placing the MCU into a sleep state, awaiting a new burst. As noted previously, during this phase, despite being in a low-power mode, the MCU displays residual consumption, which slows down the capacitor’s charging phase.

Because the voltage level barely increases, we can say that this setup (one collection and transmission every 5 min) is the minimum for the system to work. However, this rate is nearly the same as the recommended channel occupancy limit for LoRaWAN transmissions, which is about 3 min for the settings used. Therefore, we can affirm that the piezos, when utilized as harvesters, can sustain the system’s operation. Another noteworthy observation, evident from comparing the three traces, is the variable slope of the charging phase between two transmission peaks. Notably, the red trace, corresponding to a lower transmission rate compared to the blue one, exhibits a smaller slope during the initial twenty-minute observation interval. This behavior appears inconsistent with theoretical expectations, as lower transmission rates typically coincide with faster charging trends. However, this discrepancy can be attributed to the inherent instability of the vibrating setup, which may require extended intervals to stabilize. This instability is particularly evident in the latter twenty minutes, where the red trace surpasses the blue one. Despite this unexpected effect, the positive trend is still present, demonstrating the effectiveness of the charging process even in not ideal conditions.

Building upon the findings from the power consumption tests, the subsequent discussion chapter explores the implications of the results and explores potential further research and development.

## 6. Discussion

The studies performed on the proposed architecture reported interesting results. An array of five piezoelectric transducers was tested, demonstrating its capability to sustain a general low-power IoT application by harvesting energy from vibrations. In the final test campaign, which monitored the system during many transmission bursts within a 40-min timeframe, it was demonstrated that the piezoelectric transducers can provide energy autonomy for transmissions occurring at intervals of no less than 5 min. Although individual transducers generated relatively low currents, their small size and low cost make them suitable for parallel connection. This configuration can significantly increase overall harvesting efficiency, which proves useful in practical applications where high-power sensors might be integrated. In such scenarios, the ease of adding multiple transducers allows the system to harvest enough energy to support quasi real-time data transmission. Furthermore, the addition of multiple piezoelectric sensors can be beneficial, mostly in real-world environments, particularly where vibrations are not strictly sinusoidal and occur across a wide frequency range. Unlike sinusoidal functions, which are confined to specific frequencies, random vibrations span a continuous spectrum with fluctuating amplitudes and phases over time. Such vibrations are prevalent in applications such as vehicle dynamics and industrial machinery. As demonstrated in this paper, employing small piezoelectric sensors like the LDTM-028K enables easy adaptation of harvesting systems to various frequencies. By mounting multiple transducers with different free lengths (and therefore assigning specific frequency ranges to each), energy can be harvested from a diverse range of frequencies. This versatility is facilitated by the compact dimensions of these sensors and could not be obtained with the piezoelectric energy-harvesting systems analyzed up to now in the literature. Consider the scenario where there is a need to harvest energy from a car’s vibrations across various road types. Typically, with harvesters available in the literature, it is challenging to consistently extract sufficient energy because they are tuned to specific frequencies. However, by adhering to vibrational limits set by standards (e.g., ISO 16750-3 [32] for road vehicle equipment), it is feasible to extract the frequency range within which a specific car component must vibrate. By mounting multiple small piezoelectric sensors with different free lengths, one gains the flexibility to consistently extract the necessary energy. This approach ensures that regardless of the road type, sufficient energy can be harvested. Moreover, the calculation of the necessary free length becomes straightforward once an equation like (4) is available.

To this end, this paper deliberately explores the behavior of piezoelectric sensors when subjected to a frequency different from their “built-in” one, with the aim of demonstrating the feasibility of creating fully operational harvesting systems under diverse environmental conditions. The tests were indeed conducted at 50 Hz, whereas the maximum harvested energy occurs around 54–55 Hz for a fixed free length of 17 mm. Remarkably, a positive charging trend was observed despite the setup not operating under ideal conditions. This observation highlights the adaptability of these devices to harvest energy from the environment, even without being finely tuned to a specific working frequency. Additionally, the selection of the 50 Hz frequency was based on its proximity to the most prevalent frequencies in industrial vibrations. In industrial settings, vibration frequencies are often regulated according to standards such as ISO 10816 [33], which provides guidelines for evaluating machine vibration by measuring vibration severity. These standards typically present Power Spectral Density (PSD) curves, outlining a profile of acceleration versus frequency for random vibrations. These curves are valuable for easily determining the operating spectrum of industrial machinery vibrations.

The next section summarizes these insights into practical recommendations and outlines the significance of this study’s outcomes.

## 7. Conclusions

This paper aimed to demonstrate the feasibility of using commercially available piezoelectric transducers, typically used as standard sensors, to generate enough power to serve as an alternative energy source for low-power data transmission in an IoT application. The LoRaWAN architecture was chosen due to its good adaptability for low-power sensor nodes. We proposed a WSN specifically designed for low-power applications, where any sensor with dominant power consumption during a transmission burst can be integrated into the system. Piezoelectric transducers can nowadays be employed in a wide variety of environments; however, in an industrial scenario, they can easily be employed to harvest energy from many vibrating surfaces with very little cost and good scalability.

Although the proposed energy-harvesting architecture shows good results, there is room for improvement when integrating practical sensors. We plan to conduct extended day-long tests. In the current tests, observation time was limited by our vibration generation method. Cooling fans were used to manage heat buildup in the amplifier and body shaker, but this limited vibration amplitude and shortened tests.

As future work, real implementations of the solution are expected to be developed. Indeed, this work aimed at providing general-purpose results: this means that no specific sensor was chosen when conducting the tests. However, in order to fully validate the technology, a real implementation of a sensing system is expected to be carried out. To this aim, a possible scenario may foresee the implementation of an environmental monitoring solution to be deployed in a construction site. Indeed, in this application scenario, machines generating vibrations are relatively common and may be thus equipped with the piezoelectric harvesters powering the sensing platforms. Moreover, environmental monitoring proves to be an especially challenging test bench, since the measurement of air-related parameters (Carbon Dioxide, Carbon Monoxide, Particulate Matter, etc.) requires, in general, extremely power-hungry sensors.

Another exciting future development involves a monitoring sensor node with a rechargeable battery to back up the system during temporary harvesting outages. Although the storage capacitor worked during transmission bursts, its internal resistance resulted in quick energy dissipation when the harvesting source was temporally disconnected from the harvester. This limits deployment in critical scenarios like alarm systems where reliable power is essential.

Finally, another aspect that may be investigated in the near future concerns the utilization of hybrid harvesting solutions. Indeed, harvesters embedded within IoT devices are in general of small dimensions and are thus able to generate a limited amount of energy. However, more than one energy source may be present in the deployment location. For example, within the industrial context, together with vibrations, heat sources may also be available, suggesting the combination of TEGs and piezoelectric sensors. Such a promising approach, which may foresee the combination of more than one energy source, may be especially suitable in all those applications featuring a relatively high energy requirement.

## Figures and Tables

**Table 1:** Current consumption $I_{j}$ and active time $\Delta t_{A}$ for all the system components.

Component	Operating Mode		
	Active		Sleep
	$\Delta t_{A}$ (ms)	$I_{A}$ [mA]	$I_{S}\;\left[\mu A\right]$
LoRa Module	50	40	0.20
LTC3588-1	70	$1\cdot 10^{-3}$	1
ATtiny84	70	0.4	4.20
HC-SR04 & Step-up	40	~9	~0

**Table 2:** Piezoelectric sensor’s materials and their main mechanical parameters.

	Thickness [μm]	$\rho \;[g\cdot {\mathrm{cm}}^{-3}]$	Young’s Modulus (GPa)	Poisson’s Ratio
Mylar [28]	127	1.38	5.5	0.4
Ag ink [29]	10	10.49	11	0.37
PVDF [30]	28	1.78	2	0.29
Coating [31]	32	1.37	12	0.39

## Data Availability

Data are contained within the article.

## References

[1] Peruzzi G., Pozzebon A. (2020). A review of energy harvesting techniques for Low Power Wide Area Networks (LPWANs). Energies.

[2] Cappelli I., Fort A., Pozzebon A., Tani M., Trivellin N., Vignoli V., Bruzzi M. (2022). Autonomous IoT Monitoring Matching Spectral Artificial Light Manipulation for Horticulture. Sensors.

[3] Cappelli I., Parrino S., Pozzebon A., Salta A. (2021). Providing Energy Self-Sufficiency to LoRaWAN Nodes by Means of Thermoelectric Generators (TEGs)-Based Energy Harvesting. Energies.

[4] Migliorini M., Pozzebon A. Thermoelectric Generators (TEG) for the Powering of Energy-Hungry LoRaWAN-Based Sensor Nodes in Industrial Applications. Proceedings of the 2023 IEEE International Workshop on Metrology for Industry 4.0 & IoT (MetroInd4.0&IoT).

[5] Olzhabay Y., Ng A., Ukaegbu I.A. (2021). Perovskite PV Energy Harvesting System for Uninterrupted IoT Device Applications. Energies.

[6] Bruzzi M., Cappelli I., Fort A., Pozzebon A., Vignoli V. (2022). Development of a Self-Sufficient LoRaWAN Sensor Node with Flexible and Glass Dye-Sensitized Solar Cell Modules Harvesting Energy from Diffuse Low-Intensity Solar Radiation. Energies.

[7] Muscat A., Bhattacharya S., Zhu Y. (2022). Electromagnetic vibrational energy harvesters: A review. Sensors.

[8] Boisseau S., Despesse G., Seddik B.A. (2012). Electrostatic Conversion for Vibration Energy Harvesting. Small-Scale Energy Harvesting.

[9] Takhedmit H., Saddi Z., Karami A., Basset P., Cirio L. (2017). Electrostatic vibration energy harvester with 2.4-GHz Cockcroft–Walton rectenna start-up. Comptes Rendus Phys..

[10] Niu S., Wang Z.L. (2015). Theoretical systems of triboelectric nanogenerators. Nano Energy.

[11] Sezer N., Koç M. (2021). A comprehensive review on the state-of-the-art of piezoelectric energy harvesting. Nano Energy.

[12] Shirvanimoghaddam M., Shirvanimoghaddam K., Abolhasani M.M., Farhangi M., Barsari V.Z., Liu H., Dohler M., Naebe M. (2019). Towards a green and self-powered Internet of Things using piezoelectric energy harvesting. IEEE Access.

[13] Liu Y., Khanbareh H., Halim M.A., Feeney A., Zhang X., Heidari H., Ghannam R. (2021). Piezoelectric energy harvesting for self-powered wearable upper limb applications. Nano Select.

[14] Shenck N.S., Paradiso J.A. (2001). Energy scavenging with shoe-mounted piezoelectrics. IEEE Micro.

[15] Starner T. (1996). Human-powered wearable computing. Ibm Syst. J..

[16] Xu Z., Jin C., Cabe A., Escobedo D., Hao N., Trase I., Closson A.B., Dong L., Nie Y., Elliott J. (2020). Flexible energy harvester on a pacemaker lead using multibeam piezoelectric composite thin films. Acs Appl. Mater. Interfaces.

[17] Zhou Z., Zhang H., Qin W., Zhu P., Wang P., Du W. (2022). Harvesting Energy from Bridge Vibration by Piezoelectric Structure with Magnets Tailoring Potential Energy. Materials.

[18] Chen N., Jung H.J., Jabbar H., Sung T.H., Wei T. (2017). A piezoelectric impact-induced vibration cantilever energy harvester from speed bump with a low-power power management circuit. Sens. Actuators A Phys..

[19] Song Y., Yang C.H., Hong S.K., Hwang S.J., Kim J.H., Choi J.Y., Ryu S.K., Sung T.H. (2016). Road energy harvester designed as a macro-power source using the piezoelectric effect. Int. J. Hydrog. Energy.

[20] Dziadak B., Kucharek M., Starzyński J. (2022). Powering the WSN Node for Monitoring Rail Car Parameters, Using a Piezoelectric Energy Harvester. Energies.

[21] Covaci C., Gontean A. (2019). Energy harvesting with piezoelectric materials for IoT—Review. ITM Web Conf..

[22] Ali A., Ali S., Shaukat H., Khalid E., Behram L., Rani H., Altabey W.A., Kouritem S.A., Noori M. (2024). Advancements in piezoelectric wind energy harvesting: A review. Results Eng..

[23] Chen L., Xu X., Zeng P., Ma J. (2014). Integration of energy harvester for self-powered wireless sensor network nodes. Int. J. Distrib. Sens. Netw..

[24] TPA3116D2 15-W, 30-W, 50-WFilter-Free Class-D Stereo Amplifier Family with AM Avoidance. https://www.ti.com/product/TPA3116D2.

[25] LDT0-028K: LDT with Crimps Vibration Sensor/Switch. https://eu.mouser.com/datasheet/2/418/5/NG_DS_LDT_with_Crimps_A1-1130083.pdf.

[26] LTC3588-1, Nanopower Energy Harvesting Power Supply. https://www.analog.com/en/products/ltc3588-1.html.

[27] HOPERF (2018). Low Power Long Range Transceiver Module Model No.:RFM95W/96W/98W. https://cdn.sparkfun.com/assets/a/9/6/1/0/RFM95W-V2.0.pdf.

[28] Trejo M., Romero V., Hammc E., Cerda E. (2022). Lateral indentation of a thin elastic film. Soft Matter.

[29] Vasiljevic D.Z., Menicanin A.B., Zivanov L.D. (2013). Mechanical Characterization of Ink-jet Printed ag Samples on Different Substrates. Technological Innovation for the Internet of Things, Proceedings of the 4th IFIP WG 5.5/SOCOLNET Doctoral Conference on Computing, Electrical and Industrial Systems, DoCEIS 2013, Costa de Caparica, Portugal, 15–17 April 2013.

[30] Vinayaga K.K., Vasanthanathan A., Nagaraj P. (2018). Finite element modeling of smart piezoelectric beam using ANSYS®. Mater. Today Proc..

[31] Overview of Materials for Epoxy Cure Resin. https://www.matweb.com/search/datasheet.aspx?matguid=956da5edc80f4c62a72c15ca2b923494.

[32] International Organization for Standardization (2023). Road Vehicles, Environmental Conditions and Testing for Electrical and Electronic Equipment, Part 3: Mechanical Loads.

[33] International Organization for Standardization (2018). Mechanical Vibration, Measurement and Evaluation of Machine Vibration, Part 8: Reciprocating Compressor Systems.

